# Ulcerative colitis and thrombocytosis: Case report and literature review

**DOI:** 10.1097/MD.0000000000033784

**Published:** 2023-05-17

**Authors:** Yaqi Zhou, Fengqin Zhu, Dehuai Jing, Quanyi Wang, Guangxi Zhou

**Affiliations:** a Department of Clinical Medicine, Jining Medical University, Jining, Shandong, P.R. China; b Department of Gastroenterology, Affiliated Hospital of Jining Medical University, Jining Medical University, Jining, Shandong, P.R. China; c Pathology Department, Affiliated Hospital of Jining Medical University, Jining Medical University, Jining, Shandong, P.R. China.

**Keywords:** thrombocytosis, thromboembolism, ulcerative colitis

## Abstract

Ulcerative colitis (UC) is an autoimmune disease of unknown etiology, sometimes associated with anemia and thrombocytosis. Platelets (PLTs) play a role in amplifying inflammatory and immune responses in chronic inflammation. This study discusses the diagnosis and treatment of a case of UC combined with secondary thrombocytosis and reviews the relevant literature. We report an interaction between thrombocytosis and UC to raise clinicians’ awareness of this condition.

**Patient concerns:**

In the current report, we discuss the case of a 30-year-old female patient who presented with frequent diarrhea and thrombocytosis.

**Diagnosis:**

Severe UC combined with intestinal infection was diagnosed based on colonoscopy and intestinal biopsy. The patient had a PLT count >450 × 10^9^/L and was diagnosed with reactive thrombocytosis.

**Interventions and outcomes:**

The patient was discharged from the hospital in remission after receiving vedolizumab and anticoagulant treatment.

**Lessons:**

In patients with severe UC with thrombocytosis, clinicians should pay attention to PLTs promoting inflammatory progression, as well as screening for venous thromboembolism risk and prophylactic anti-venous thromboembolism therapy at the time of dosing to avoid adverse effects.

## 1. Introduction

Ulcerative colitis (UC) is a relapsing inflammatory bowel disease (IBD) characterized by chronic and recurrent gastrointestinal inflammation. The pathogenesis of UC is complicated and is still unclear. The main clinical manifestations are diarrhea, bloody, and purulent stools. In addition, UC is often accompanied by anemia and thrombocytosis.

Platelets (PLTs) are not only involved in hemostasis and thrombosis, but they are also important in amplifying inflammatory and immune responses in chronic inflammation.^[1]^ It has been reported that the exacerbation of IBD is accompanied by increased PLT count.^[2]^ The blood is in a hypercoagulable state, and the risk of thrombotic disease in patients increases accordingly.^[3]^ Here, we report a case of UC presenting as thrombocytosis, which has not been reported in published studies, and provide a review of the relevant literature.

## 2. Case presentation

A 30-year-old female patient presented with a half-month history of diarrhea (3–4 times per day), which gradually evolved into an increase of diarrhea (5–6 times per day), accompanied by nausea and vomiting. Her stools were watery, mixed with a small amount of blood, and she had no abdominal pain. She had been admitted to a local hospital and was discharged in remission after anti-infection and other treatments. On July 2, 2021, the patient developed a fever with a maximum temperature of 38.9°C. The fever was followed by diarrhea but without abdominal pain. After symptomatic treatment at the local clinic, she still had a recurrent fever.

For further evaluation and treatment, the patient came to our hospital (the affiliated hospital of Jining Medical University) on July 6. She had a previous history of hemorrhoids and cesarean delivery, with no history of drug abuse or food allergy. The physical examination suggested that she had an anemic appearance with pale lips and no other special signs.

**Figure 1.**
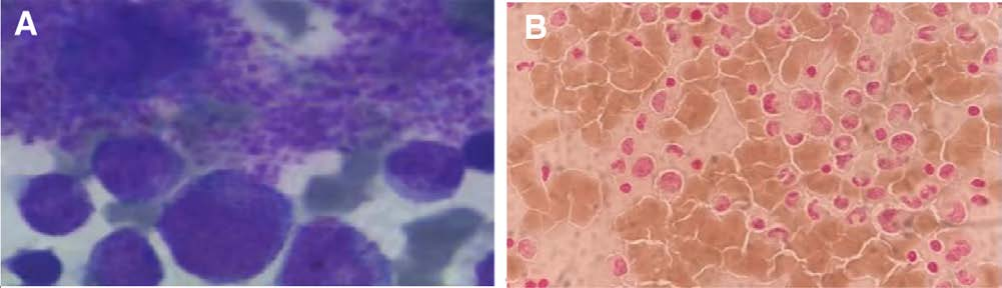
The bone marrow smear findings. (A) Thrombocytosis, iron staining suggests (B) intracellular iron deficiency.

The patient was admitted to the outpatient department for routine blood tests. The results are shown in Table 1, indicating an elevated PLT level of 584 × 10^9^/L and decreased hemoglobin of 79 g/L (Table 1). Therefore, the patient was admitted to the Department of Hematology. Red and white blood cells were found, but no Shigella or Salmonella was cultured in her stool examination. The blood workup revealed elevated C-reactive protein (Table 1). We initially considered that the patient had infectious diarrhea. At the same time, the increased PLTs may be caused by inflammation. The bone marrow examination suggested intracellular and extracellular iron deficiency, active hyperplasia of metamyelocytes, and iron staining suggesting intracellular and extracellular iron deficiency (Fig. 1). The results of the coagulation function, urine, liver function, kidney function, and antinuclear antibody spectrum were all normal.

**Table 1:** The result of blood routine examination.

Date	WBC (×10^9^/L)	HGB (g/L)	PLT (×10^9^/L)	RBC (×10^12^/L)	CRP (g/L)
July 6, 2021	19.07	79	584	4.47	102
July 10, 2021	15.43	64	526	3.55	166
July 21, 2021	8.72	85	392	3.84	21.26
July 24, 2021	7.85	82	527	3.55	–
August 7, 2021	8.77	97	515	3.70	1.88
September 26, 2021	7.50	126	346	4.37	0.00

CRP = C-reactive protein, HGB = hemoglobin, PLT = platelet, RBC = red blood cell, WBC = white blood cell.

**Figure 2.**
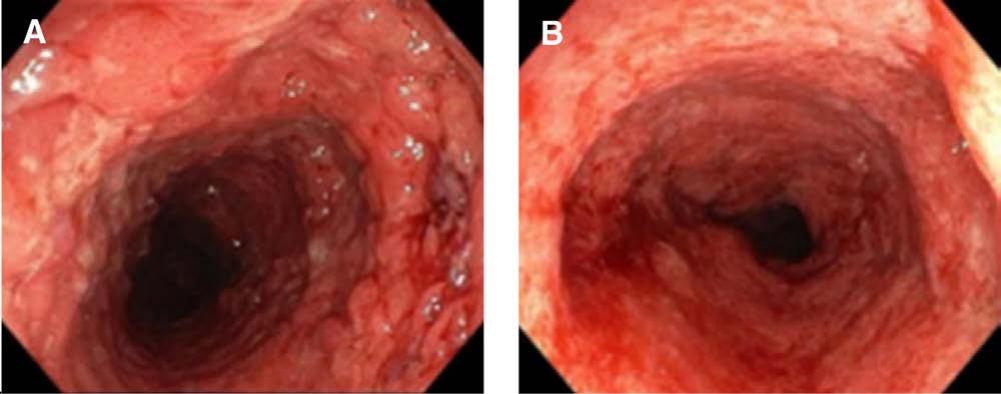
Colonoscopy images. The images revealed hyperemia and edema of the (A) transverse colon and (B) rectal mucosa, blurred vascular texture, and local shallow ulceration.

**Figure 3.**
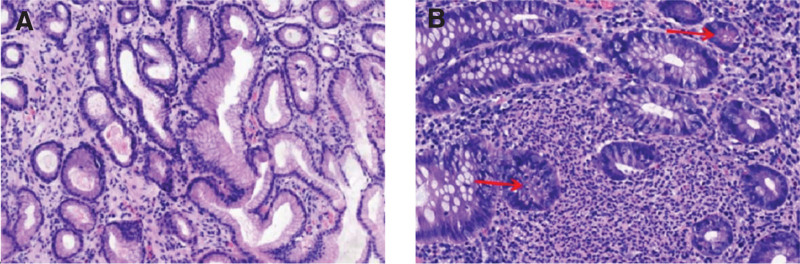
Pathological pictures show chronic inflammation of (A) gastric body mucosa, ileocecal mucosal crypt abscess formation (arrow), and (B) neutrophil infiltration.

**Figure 4.**
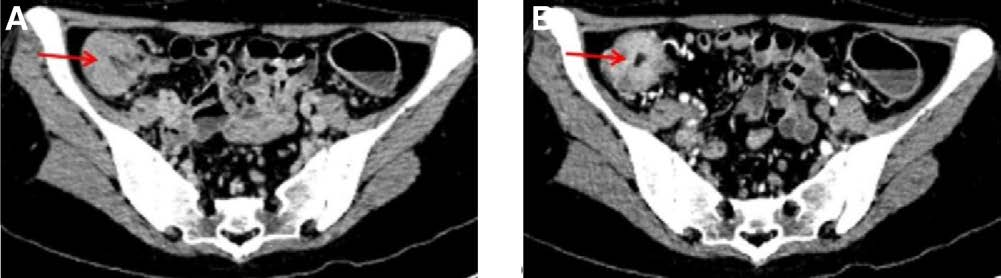
Abdominal enhanced CT images. The images show thickening of the (A) ileocecal and (B) colorectal walls (arrows). CT = computed tomography.

The patient was initially diagnosed with secondary thrombocytosis, infectious diarrhea, and iron deficiency anemia. Hence, she received intravenous cefoperazone/sulbactam (3 g every 8 hours) for anti-infection treatment, PLT apheresis to reduce PLTs, plasma infusion, and iron supplements. However, she still had repeated fever and persistent diarrhea. PLT (526 × 10^9^/L) did not decrease after reexamination on July 10. She discontinued cefoperazone/sulbactam. After a combination of oral vancomycin (0.25 g every 6 hours) and intravenous tigecycline (50 mg every 12 hours) for stronger anti-infective treatment, she no longer had a fever but still had recurrent diarrhea with a small amount of blood mixed in the stool. Thus, further gastrointestinal endoscopes were done on July 13. Colonoscopy revealed hyperemia and edema of the colorectal mucosa, blurred vascular texture, and local shallow ulceration (Fig. 2). Intestinal mucosal biopsy exhibited acute and chronic inflammatory cell infiltration, necrosis, and crypt abscess formation. Gastric mucosal biopsy showed chronic inflammation of the superficial mucosa (Fig. 3). The patient’s colonoscopy findings met the diagnostic criteria for UC. In addition, Gram-positive cocci were found in fecal culture. Her venous thromboembolism (VTE) risk assessment score is 1, which was low risk. Bilateral lower extremity ultrasound testing revealed no deep venous thrombosis. Abdominal enhanced computed tomography scans showed thickening of ileocecal and colorectal intestinal walls (Fig. 4).

Based on the clinical manifestations, colonoscopy images, pathological findings, and computed tomography scans, we concluded that UC with infection was the most likely diagnosis. On July 13, the patient was transferred to the Department of Gastroenterology for continued treatment. We administer mesalazine sustained-release granules (2 g twice per day), intravenous cefoperazone/sulbactam (3 g every 8 hours), and oral vancomycin (0.5 g every 12 hours) for anti-infection. She had high PLTs, probably in a hypercoagulable state. For thromboembolism prophylaxis, she was anticoagulated with nadroparin (0.4 mL per day). On July 23, she received 300 mg of intravenous vedolizumab. When the patient was discharged from the hospital, she had no fever and had stools 2 to 3 times a day without pus or blood. However, the reexamination of the blood routine showed that PLTs reached 527 × 10^9^/L, which was still very high (Table 1).

Following, the patient was administrated 300 mg vedolizumab in our hospital on August 6 and September 26, respectively. The results of the repeat routine blood tests showed that her inflammatory indicators were lower than before, her PLTs dropped to normal, and her stool was normal. Her VTE risk assessment score is 0.

## 3. Discussion

IBD refers to a chronic nonspecific intestinal inflammatory disease, mainly including UC and CD.^[4]^ The clinical manifestations of UC include abdominal pain, diarrhea, and bloody purulent stool, with lesions primarily affecting the colonic mucosa and submucosa.^[5]^ Numerous extraintestinal complications, such as anemia and thrombosis, are related to UC.^[6–8]^ The incidence of UC has increased significantly in Asia over the past few decades, greatly affecting patients’ quality of life. It has a complicated pathophysiology, and its cause is unknown.^[9–12]^ Numerous studies have shown that PLTs play an important role in the development of UC and the associated complications. Tissue damage from surgery, infection, cancer, or chronic inflammation drives the body to produce a range of pro-inflammatory cytokines and other inflammatory mediators, which can cause aberrant bone marrow thrombopoiesis.^[13]^ In 1968 Morowitz first presented the first evidence of PLT abnormalities in patients with IBD, describing increased PLTs in patients with worsening clinical activity. Another way, IBD causes reactive thrombocytosis in patients.^[14]^ During the active phase of the disease, reactive thrombocytosis (RT) is defined as > PLTs 450 × 10^9^/L.^[15]^ PLT-related parameters (such as mean platelet volume, platelet distribution width, and thrombocytocrit) can be used to predict disease activity in IBD.^[16]^

Currently, a growing number of studies support the important role of PLTs in amplifying inflammation in chronic inflammatory diseases.^[17]^ PLTs are activated when they pass through small veins in the inflamed intestinal wall.^[16]^ UC is characterized by changes in PLT number and function, such as thrombocytosis and enhanced PLT activation. Thrombocytosis has now been established to correlate with disease activity and severity in UC.^[18]^ During the active phase of UC, activated PLT release factors, notably CD40L. They bind and activate receptors on circulating immune cells or endothelial cells, amplify inflammatory responses, promote thrombosis, and aggravate intestinal mucosal injury.^[19,20]^ In our patient, she had elevated PLT and no PLT abnormalities on bone marrow examination, then we considered reactive (secondary) thrombocytosis caused by UC. Increased PLTs exacerbated the inflammation of UC. However, we only did 1 blood cell collection to reduce PLTs, and the PLT was still high. When she was discharged from the hospital, which may delay the resolution of inflammation. We should reflect on the risk of PLT-amplified inflammatory responses while actively treating UC.

PLTs play an important role in hemostasis and thrombosis. PLTs form a thrombus at the site of vascular injury, including vascular injury, atherosclerotic plaque rupture, and chronic inflammatory disease. They have long been a major therapeutic target for thromboembolism prevention.^[21]^ Thromboembolism is a specific extraintestinal complication of IBD. Compared with the general population, patients with IBD have a nearly 3-fold increased risk of developing thromboembolism, mainly in the venous circulation.^[22,23]^ It has been reported that the increased risk of thromboembolism is related to the overexpression of CD40L in PLTs, which may be the only source of CD40L in IBD patients.^[6,24,25]^ Patients hospitalized with IBD have a higher risk of VTE and VTE-related mortality compared with hospitalized patients without IBD.^[26]^ Moderately to severely active UC is an important risk factor for VTE. Moreover, VTE is related to the degree of colon involvement, and 71% of UC patients with VTE have extensive colon involvement.^[27]^ Administration of thromboprophylaxis drugs during hospitalization reduces the risk of VTE after hospitalization.^[28]^ Therefore, for moderate-to-severe hospitalized patients without severe bleeding, VTE risk screening and prophylactic anti-VTE treatment are recommended.^[29]^ In the present case, we gave the patient anticoagulation to prevent VTE, and she had not developed VTE before discharge.

We found that the patient had iron deficiency anemia, which has been reported to be a common extraintestinal manifestation of IBD, emerging in more than one-third of the patients.^[30]^ The most prevalent cause of anemia in IBD is iron deficiency anemia, with approximately 77.5% of newly diagnosed IBD patients exhibiting iron deficiency.^[8,31]^ Notably, thrombocytocrit is associated with certain markers of iron deficiency (e.g., soluble transferrin receptor and hemoglobin), and some mechanisms associated with iron deficiency are associated with PLT overproduction.^[17]^ Iron deficiency may be an important mechanism of RT, and iron supplementation therapy in patients with IBD anemia normalizes PLT count and reduces PLT activity.^[32,33]^ In our case, giving the patient iron supplementation was also beneficial in the treatment of RT.

We initially diagnosed the patient with infectious diarrhea. The clinical manifestations of active UC and infectious colitis, such as abdominal pain, diarrhea, and bloody purulent stools, have common features, so it is not easy to differentiate between UC and infectious diarrhea. Infectious diarrhea often has an epidemiologic history, such as a history of travel or eating unclean food. Common inflammatory pathogens include *Shigella, Campylobacter, Salmonella*, and *Clostridium difficile*.^[34]^ Colonoscopy and intestinal mucosal biopsy often show specific manifestations of viral, bacterial, or parasitic infection. Although UC lacks a gold standard for diagnosis, the patient’s colonoscopy and mucosal biopsy findings are consistent with the primary basis for the diagnosis of UC. And severe UC presents with >6 bloody stools per day, evidence of toxicity in the form of fever, tachycardia, anemia, or elevated erythrocyte sedimentation rate.^[35]^ In addition, PLT is considered a marker to distinguish IBD from infectious diarrhea, in which thrombocytosis is not common.^[36]^ Therefore, based on the above evidence, we ruled out the diagnosis of infectious diarrhea, preferring that the patient had severe UC complicated with an intestinal infection. Meanwhile, the patient’s stool culture only found gram-positive cocci. We consider the invasion of gram-positive cocci due to impaired intestinal mucosal barrier function.^[37]^ Gram-positive cocci such as *Staphylococcus aureus* can adhere to intestinal epithelial cells, which is closely related to the occurrence and progression of UC.^[38]^

Despite recent advances in IBD therapeutic, a high proportion of patients remain refractory to conventional treatment (e.g., sulfasalazine, aminosalicylates, cortisols, and immunosuppressants), so treatment options for moderately to severely active UC remain limited.^[39]^ Anti-TNF-α agents such as infliximab, adalimumab, and golimumab, as well as anti-integrin agents such as vedolizumab, are usually the primary therapy for moderate-to-severe UC, and current evidence supports infliximab or vedolizumab as first-line therapy for moderate-to-severe UC,^[39,40]^ but anti-TNF therapy appears to increase the risk of VTE in inflammatory disease. Infliximab can induce antiphospholipid antibodies, which can cause coagulation abnormalities by damaging endothelial and PLT functions, and then increase the risk of VTE.^[41]^ Moreover, infliximab may cause opportunistic infections and increase the risk of malignancies. Vedolizumab is an entero-selective preparation with relatively high safety, and by reviewing the relevant data, we did not find any reports of vedolizumab being associated with thrombosis. Studies have shown that vedolizumab is effective as both induction and maintenance therapy in moderately to severely active UC, the disease remission rate is higher than that of TNF antagonists,^[42,43]^ and does not significantly increase the risk of serious systemic opportunistic infections.^[44]^ Additionally, activated PLTs are active coconspirators of inflammation and tissue damage and represent a significant risk factor for the amplification of intestinal inflammation. They are sought to be plausible targets for specific therapeutic interventions. Studies have found that rupatadine is a PLT-activating factor receptor antagonist, which treats UC by inhibiting PLT-activating factor-related pathways. Some antagonistic mAbs to CD40, such as ch5D12, are still in the experimental stage. In our case, the patient was treated with vedolizumab, resulting in a favorable prognosis.

## 4. Conclusion

For UC patients presenting with thrombocytosis, clinicians should pay attention to diagnosis and treatment. Thrombocytosis promotes thrombus formation and inflammation progression, which should be a wake-up call if a patient has thrombocytosis. The followings are the key findings of this case: for patients with moderate-to-severe UC, screening for VTE risk (e.g., D-dimer, lower extremity venous ultrasound, etc) and prophylactic anti-VTE therapy should be performed; anemia-related indicators should be monitored; in the presence of thrombocytosis, medications for UC therapy should be attention.

## Author contributions

**Conceptualization:** Yaqi Zhou, Fengqin Zhu.**Data curation:** Dehuai Jing, Guangxi Zhou.

Funding acquisition: Guangxi Zhou.

Investigation: Quanyi Wang.

Writing – original draft: Yaqi Zhou.

Writing – review & editing: Guangxi Zhou.
